# Liquid-Metal-Mediated Electrocatalyst Support Engineering toward Enhanced Water Oxidation Reaction

**DOI:** 10.3390/nano12132153

**Published:** 2022-06-23

**Authors:** Guyue Bo, Peng Li, Yameng Fan, Qiang Zhu, Linlin Xia, Yi Du, Shi Xue Dou, Xun Xu

**Affiliations:** 1 Institute for Superconducting & Electronic Materials, Australian Institute for Innovative Materials, University of Wollongong, Wollongong, NSW 2522, Australia; gb029@uowmail.edu.au (G.B.); yf645@uowmail.edu.au (Y.F.); yi_du@buaa.edu.cn (Y.D.); shi@uow.edu.au (S.X.D.); xun@uow.edu.au (X.X.); 2 Electron Microscopy Center, University of Wollongong, Wollongong, NSW 2500, Australia; qzhu@uow.edu.au; 3 School of Automation Engineering, Northeast Electric Power University, Jilin 132012, China; xiall521@neepu.edu.cn; 4 School of Physics and BUAA-UOW Joint Research Centre, Beihang University, Beijing 100191, China

**Keywords:** GaInSn eutectic alloy, aluminum oxide, manganese oxide, oxygen evolution reaction, electrocatalysis

## Abstract

Functional and robust catalyst supports are vital in the catalysis field, and the development of universal and efficient catalyst support is essential but challenging. Traditional catalyst fabrication methods include the carbonization of ordered templates and high−temperature dehydration. All these methods involve complicated meso−structural disordering and allow little control over morphology. To this end, a eutectic GaInSn alloy (EGaInSn) was proposed and employed as an intermediate to fabricate low−dimensional ordered catalyst support materials. Owing to the lower Gibbs free energy of Ga_2_O_3_ compared to certain types of metals (e.g., Al, Mn, Ce, etc.), we found that a skinny layer of metal oxides could be formed and exfoliated into a two−dimensional nanosheet at the interface of liquid metal (LM) and water. As such, EGaInSn was herein employed as a reaction matrix to synthesize a range of two−dimensional catalyst supports with large specific surface areas and structural stability. As a proof−of-concept, Al_2_O_3_ and MnO were fabricated with the assistance of LM and were used as catalyst supports for loading Ru, demonstrating enhanced structural stability and overall electrocatalytic performance in the oxygen evolution reaction. This work opens an avenue for the development of functional support materials mediated by LM, which would play a substantial role in electrocatalytic reactions and beyond.

## 1. Introduction

Electrochemical energy conversion and storage are important solutions in the pursuit of attaining the green utilization of renewables in the form of chemical energy. In these areas, electrocatalytic water splitting has long been considered an indispensable method for producing green hydrogen (H_2_) with the use of renewable electricity [1,2,3,4]. Unfortunately, the question of how to develop functional and robust electrocatalysts with excellent overall performance remains a scientific issue. Most commercial electrocatalysts are usually produced by dispersing active metal species in or on a support material to maximize the overall catalyst stability, durability, and catalytic activity. Furthermore, the electronic structure of an electrocatalyst can be well modulated through metal−support interaction, which can be accessed through a wise choice of a range of different types of support materials. Support materials are therefore rather significant in the development of functional electrocatalysts—a consensus that has been widely accepted within the electrocatalysis community, as an increasing number of efforts concentrated on support materials have been published very recently [5,6,7,8,9,10]. Most of these works typically focus on the tuning of metal−support interaction, while studies on the design and fabrication of functional support materials are rarely reported. Meanwhile, traditional catalyst support fabrication typically relies on hydrothermal reactions, template methods, high-temperature dehydration, etc., which involve complicated meso−structural disordering and have morphologies which are difficult to control [11,12,13,14]. The development of reliable production methods for catalyst supports is urgently needed and has many scientific implications.

Gallium-based liquid metals have attracted increasing research attention in the field of stretchable electronics, self−healing [15,16,17,18,19] and reconfigurable devices [20], functional binders [21,22,23,24], and electrocatalysis [25,26] owing to their outstanding electrical conductivity, room−temperature fluidic ability, easily tunable metallic components, etc. In particular, Zavabeti et al. reported that eutectic gallium alloys can be easily mixed with a range of transition metals and used to synthesize a variety of transition metal oxides [27,28,29,30]. In the meantime, we found that lots of catalyst supports that play significant roles in thermal catalysis and electrocatalysis are usually fabricated via conventional methods such as high−temperature decomposition and chemical vapor deposition. The specific surface areas and robust structure stability of the supports are not necessarily well sustained through such harsh reaction conditions. Therefore, finding an alternative catalyst support fabrication strategy with mild reaction conditions and well−modulated structures is essential.

In the present work, gallium−based liquid metal was employed for the synthesis of δ−Al_2_O_3_and MnO, in which the LM serves as a reaction environment and can be repeatedly used after proper and easy separation, featuring low−cost, readily available, and environmentally friendly. This method represents a novel way to produce δ−Al_2_O_3_ and MnO precursors at room temperature. The precursors can be generated from the interface of EGaInSn droplets with H_2_O, and then, the product can be collected after being sintered in a furnace. This low−level aggregation of δ−Al_2_O_3_ and MnO exhibits quasi−two−dimensional structures with a large surface area, as well as thermal stability, which provides an opportunity for the fabrication of catalyst supports. This work investigated the electrocatalytic performance of δ−Al_2_O_3_ and MnO against the performance of Ru−loaded supports to demonstrate that Al_2_O_3_ and MnO are versatile and advantageous catalyst supports and that Ru can be loaded to improve performance. A detailed analysis was shown for a better understanding of the performance of this kind of material.

## 2. Experimental Section

### 2.1. Catalyst Support Fabrication (Al_2_O_3_ and MnO)

Eutectic GaInSn alloy was prepared by co−melting a particular weight amount of Ga (65%), In (24%), and Sn (11%). In the preparation of Al_2_O_3_ support, EGaInSn (g) and Al foil (g) were ground in an Argon−filled glove box where the oxygen level and water level were controlled at levels lower than ~2 ppm. The grinding process usually takes 30 min to fully and completely finish the alloying process, and the alloy surface becomes shiny and metallic. For concentrations exceeding 20% aluminum, solid Al lumps were observed, which means the amount of aluminum was excessive. In the subsequent step, nearly 2 g of the as−prepared Ga−Al alloy was transferred into a glass vial. Then, 2 mL of deionized (DI) water was rapidly added to the glass vials (volume 7 mL) when the glass vial was removed from the Argon−filled glovebox. The amount of DI water needed to cover the metal droplet surface to maximize the interfacial reaction area. The reaction occurred immediately when DI water was added, and then continued for nearly 30 min. On the surface of EGaInSn droplets, 2D nanosheets were formed. The hydrogen bubbles instantly formed on the surface of droplets and accordingly, the metal oxide nanosheets were exfoliated. To obtain a pure sample, the sample was allowed to stand for 15 min. The suspension was transferred to an oven and dried at 80 °C overnight to obtain the precursor Al(OH)_3_. Finally, the as−prepared Al(OH)_3_ was calcined at 1000 °C (ramping rate of 1 °C min^−1^) in a Muffle furnace for 15 h to obtain the Al_2_O_3_. The metals Ga, In, Sn, Al, and Mn were purchased from Sigma Aldrich and used directly without further purification.

In terms of MnO synthesis, 2 g of EGaInSn and 0.066 g of manganese powder were ground in an Ar−filled glovebox for 15 min to form a uniform and skinny alloy. Then, the formed alloys were transferred to a vial and moved from the glove box, during which, the water was added instantly. After the 30 min reaction and then being stewed for 15 min, the suspension was dried at 100 °C overnight. Finally, the MnO was obtained after calcination at 400 °C in a Muffle furnace for 4 h.

### 2.2. Physical Characterization:

Powder X−ray diffraction (XRD) patterns of the as−prepared samples were tested by using a PANalytical Empyrean X−ray diffraction meter with Cu Kα radiation (λ = 0.15406 nm) from 20° to 80°. For XRD measurement, samples were ground with a mortar and pestle and then transferred onto a sample holder. The surface morphologies and electronic structures of the as−prepared samples were detected using scanning electron microscopy (SEM) and transmission electron microscopy (TEM, JEOL−2010). Gatan microscopy 2.32 was used to process the captured images. X−ray photoelectron spectroscopy was performed using a Thermo Fisher KAlpha system photoelectron spectrometer (100 Analyser, SPECS, Berlin, Germany, Alka X-Rays).

### 2.3. Electrochemical Characterization

Electrochemical measurements were carried out on a three−electrode setup (PINE Research Instrument Inc. Durham, NC, USA). A glass carbon electrode, a 1 M Hg/HgO electrode, and a graphite rod were used as the working electrode, reference electrode, and counter electrode, respectively. For the preparation of catalyst ink, a 2.0 mg sample was dispersed into a 500 µL mixed solution that contained 16 µL of Nafion solution, 100 µL of isopropanol, and 384 µL of DI water and sonicated for 4 h to form a homogeneous ink. Then, 10 µL of as−prepared catalyst ink was dropped onto the GC electrode (mass loading 0.204 mg cm^−2^) and dried at room temperature. A multichannel potentiostat (VSP-300, BioLogic Science Instrument) collected all the electrochemical data. For the test of OER, cyclic voltammetry was collected at a sweeping rate of 50 mV s^−1^. Linear sweep voltammetry (LSV) was recorded at the scan rate of 5 mV s^−1^ with a 1600 rpm rotating speed.

## 3. Results and Discussion

**Figure 1:**
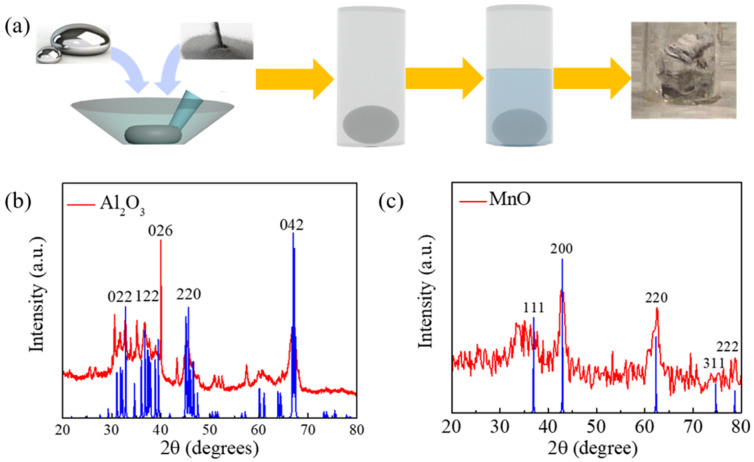
(**a**) Schematic illustration of the fabrication process of δ−Al_2_O_3_ and MnO mediated by eutectic GaInSn alloy. XRD patterns of the δ−Al_2_O_3_ (**b**) and MnO (**c**).

The catalyst support was synthesized successfully via a solution reaction followed by calcination, in which liquid metal is served as a reaction mediator. Figure 1a shows the schematic illustration of the synthetic procedure, of which the details can be found in the Experimental Section. Specifically, a certain amount of the metals (Al or Mn) and the commercial EGaInSn were ground to form uniform alloys in which the weight ratio of Al or Mn was kept at 20%. The formed alloy droplets were transferred to glass vials in the presence of DI water, and the reaction went ahead at 100 °C for 0.5 h to obtain the precursors. Due to the Gibbs free energy (ΔG_f_) for Al_2_O_3_ being lower than that of Ga_2_O_3_, a skinny layer of Al_2_O_3_ was instantly generated at the surface of the alloyed droplets [21,24,25]. In the presence of water droplets, the metal hydroxides were formed as a white aerogel, and the unreacted LM could be recycled for further utilization, indicating that the LM principally served as a reaction matrix and was not involved in the reaction. The metal oxide supports could be obtained accordingly after the as-prepared metal hydroxides’ sintering. The X−ray diffraction patterns of the as−prepared δ−Al_2_O_3_ and MnO are shown in Figure 1b−c. The main diffraction peaks located at 33.1°, 36.8°, 39.7°, and 67.1° could be well indexed to the (022), (122), (026), and (042) planes of δ−Al_2_O_3_ phase (PDF: 00−046−1131). In addition, no impurity peaks could be observed from the XRD patterns, again suggesting the successful fabrication of the pure δ−Al_2_O_3_ phase. In terms of the XRD pattern of MnO in Figure 1c, the diffraction peaks at 36.7°, 42.7°, and 62.1° corresponded to the (111), (200), and (220) face of MnO (PDF: 04−006−0700), also implying the formation of pure MnO.

**Figure 2:**
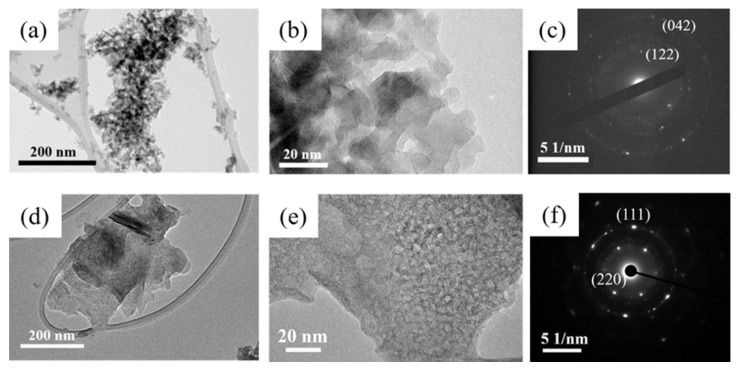
(**a**,**b**) TEM images of the as-prepared δ−Al_2_O_3_. (**c**) SAED pattern of δ−Al_2_O_3_. (**d**,**e**) TEM images of the as−prepared MnO. (**f**) SAED pattern of MnO.

Figure 2 presents the transmission electron microscopy (TEM) images of δ−Al_2_O_3_ and the MnO nanosheets. Apparently, the sheet-like morphology with sizes ranging from 20 nm to 100 nm for δ−Al_2_O_3_ is presented in Figure 2a,b, indicating the successful fabrication of a quasi−two−dimensional support. In addition, the (122) and (042) lattice planes in the selected area electron diffraction (SAED) patterns (Figure 2c) corresponded well with the two planes in the XRD pattern, again implying the formation of δ−Al_2_O_3_. For the prepared MnO support, the morphology of MnO featured a round−like nanosheet with a size of 200−300 nm. Furthermore, the (111) and (220) plane in the SAED pattern of MnO (Figure 2f) also corresponded well with the peaks at 36° and 43° of the XRD pattern, manifesting the generation of MnO support.

**Figure 3:**
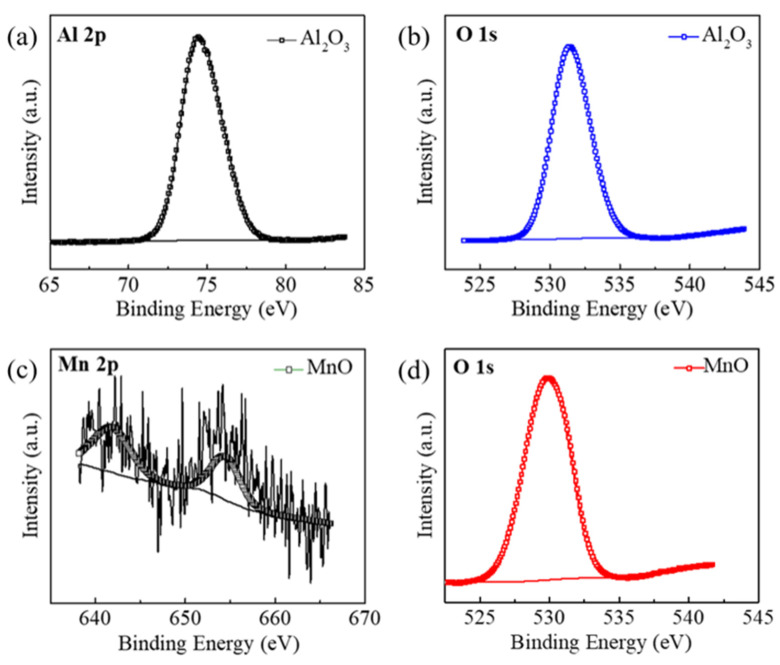
(**a**,**b**) XPS spectra of Al 2p and O1s for δ−Al_2_O_3_. (**c**,**d**) XPS spectra of Mn 2p and O 1s for MnO.

Furthermore, X−ray photoelectron spectroscopy (XPS) measurements were taken to characterize the chemical composition of the as−prepared supports. The Al 2p spectrum, as shown in Figure 3a, exhibited a prominent peak at the binding energy of 74.47 eV, which could be attributed to the oxidation state (+3) of Al. Meanwhile, the O 1s spectra in Figure 3b exhibited a major peak at 532.2 eV, which could correspond to a −2 state. The oxidation states of Al and O based on the analysis of Al 2p spectra and O 1s spectra validated the formation of an Al_2_O_3_ support. The Mn 2p spectra in Figure 3c exhibited two peaks that could be attributed to Mn (641.72 eV and 654.7 eV), also implying the formation of a MnO support.

Based on the above−mentioned discussions, the support growth principle can be reasonably deduced to demonstrate the following reaction equations:

2Al + 6H_2_O →2Al(OH)_3_ + 3H_2_ (1)

2Al(OH)_3_ →Al_2_O_3_ + 3H_2_O (2)

Mn + 2H_2_O → Mn(OH)_2_ + H_2_ (3)

Mn(OH)_2_ → MnO + H_2_O (4)

The LM-assisted interface reactions between metal and water droplets at intermediate temperature give rise to the formation of metal hydroxides, which are described in Equations (1) and (3). Subsequently, the annealing process at high−temperature conditions promotes the phase transformation from metal hydroxides to metal oxides, leading to the generation of metal oxide supports.

**Figure 4:**
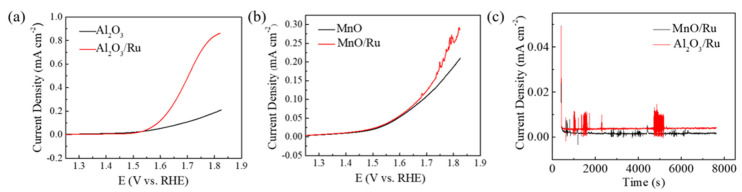
(**a**) LSV curves of δ−Al_2_O_3_ and δ−Al_2_O_3_/Ru. (**b**) LSV curves of MnO and MnO/Ru. (**c**) Chronoamperometry (CA) curve of δ−Al_2_O_3_/Ru and MnO/Ru.

Next, we discuss the role of metal oxides as supporting materials in electrocatalytic reactions, which is illustrated in Figure 4. Accordingly, the as−prepared metal oxides were used as supports to immobilize metal nanoparticles to verify their applicability. The electrocatalytic performance of δ−Al_2_O_3_, δ−Al_2_O_3_/Ru, MnO, and MnO/Ru toward oxygen evolution reaction was measured in a 1.0 M KOH solution via a three−electrode system (Experimental Section). Compared with pure δ−Al_2_O_3_ and MnO supports, the Al_2_O_3_/Ru and MnO/Ru exhibited much lower overpotentials, indicating the as−prepared metal oxide supports could promote the oxygen evolution reaction via the immobilization of Ru. As shown in Figure 4a, δ−Al_2_O_3_ can promote the oxygen evolution capability by Ru loading. The LSV curves of MnO before and after Ru loading (MnO/Ru) demonstrated that MnO can be used as a catalyst support (Figure 4b). To evaluate the durability of δ−Al_2_O_3_/Ru and MnO/Ru for OER, CA tests were conducted in 1.0 M KOH at the potential of 0.7 V. Both δ−Al_2_O_3_/Ru and MnO/Ru exhibited decent durability because no apparent current decay could be observed within the tested period of time, further suggesting that as−prepared δ−Al_2_O_3_ and MnO contribute to enhanced catalytic activity and durability.

## 4. Conclusions

In summary, we demonstrated a sustainable method to synthesize (δ−Al_2_O_3_ and MnO) catalyst supports via the mediation of EGaInSn liquid metals. The precursor of a metal hydroxide nanosheet can be formed on the liquid metal/water interface in the presence of water droplets at room temperature, and these can be easily exfoliated and transformed into metal oxide supports via a simple annealing process. Furthermore, the quasi-two-dimensional δ−Al_2_O_3_ and MnO with high specific surface areas were employed as catalyst supports, whereby the structural stability and oxygen evolution kinetics were improved through the immobilization of Ru nanoparticles. This work not only offers an easy quasi−two−dimensional catalyst support fabrication method mediated by LM but also demonstrates its vital applications in electrocatalytic reactions.

## Data Availability

The data present in this study are available on request from the corresponding author.
